# LOINC implementation approaches in academic medical research centers – results from a survey of CTSA sites

**DOI:** 10.1017/cts.2025.10151

**Published:** 2025-09-29

**Authors:** Rachel L. Richesson, Thomas R. Campion, Boyd M. Knosp, David A. Hanauer

**Affiliations:** 1 Department of Learning Health Sciences, University of Michigan Medical School, Ann Arbor, MI, USA; 2 Clinical & Translational Science Center, Weill Cornell Medical College, New York, NY, USA; 3 Roy J. and Lucille A. Carver College of Medicine and Institute for Clinical and Translational Science, University of Iowa, Iowa City, IA, USA

**Keywords:** Informatics, data standards, research infrastructure, translational science, laboratory data

## Abstract

Reference standards are vital for harmonizing heterogeneous clinical data for research, but little is known about their implementations or costs. We surveyed NIH Clinical and Translational Science Awards (CTSA) hubs to assess operational dimensions of institutional implementation, maintenance, and use of the Logical Observation Identifiers, Names, and Codes (LOINC) standard. Respondents (*n* = 19,30%) exhibited substantial variability in approaches to implementation. Differences in number and training of staff and frequency of mapping updates make it difficult to estimate costs and comparability of data across sites. CTSA and other multi-site research can benefit from operational definitions and recommended processes for LOINC implementation.

## Background

The Logical Observation Identifiers Names and Codes (LOINC) reference coding system is a mature and internationally recognized standard for laboratory and clinical observations [1]. The current version of LOINC contains more than 104,000 clinical observation and laboratory codes, and the database is continually expanding to accommodate requests for new codes [2]. As a comprehensive and highly detailed coding system, LOINC provides a reference standard terminology to harmonize multi-site data for networked research and is included in most common data models, including OMOP (Observational Medical Outcomes Partnership), PCORnet (Patient Centered Outcomes Research Network), and ENACT (Evolve to Next-Gen Accrual to Clinical Trials). Additionally, LOINC is required in the US Core Data for Interoperability (USCDI) [3] for coding laboratory tests (and many other types of observations in notes and survey questions) for clinical data exchange in Assistant Secretary for Technology Policy - Office of the National Coordinator for Health IT (ASTP-ONC) certified EHR systems and, by extension, can support “real-world data” research.

Because LOINC was developed independently and somewhat disconnected from commercial laboratory systems, the implementation and use of LOINC requires mapping local (institutional) codes from individual clinical laboratory systems to LOINC. Historically, clinical laboratories have had difficulty creating these mappings [4] and anecdotal evidence suggests that LOINC is still not widely adopted in clinical settings. There are multiple reasons for this, including the magnitude and complexity of the standard and frequency of updates [5]. Additional barriers include costs for implementation and maintenance (due to the need for oversight by subject matter experts), lack of practical implementation guidance, and limited business cases (for both clinical and research contexts) for interoperability and data exchange using LOINC.

Over the past two decades, multiple studies have explored the consistency of LOINC mapping across different settings, reporting high variability of code assignment between sites [6–8] and manufacturers [9]. A study reported this year found inconsistencies in 17% of 962 LOINC codes linked to 162 tests across seven institutions in Korea [6]. These studies use different approaches (e.g., Regenstrief LOINC Mapping Assistant, RELMA [10], UMLS, institutional, or research network-specific defined mappings) for mapping local tests to LOINC codes and for validation. This variation suggests an unaddressed need for guidance on how (e.g., single versus multiple reviewers; adjudication) and who (e.g., pathologist, laboratorian, non-pathologist physician) should create and validate mappings).

Despite these challenges, academic medical centers and health systems are increasingly expected to encode and share their lab data using LOINC in order to participate in research initiatives, including multi-institutional networks, and Clinical and Translational Science Award (CTSA) hubs are expected to have LOINC codes to support institutional and national research activities. Although the use of standards is a critical component of research maturity, an operational definition for an “organizational implementation” of LOINC does not exist [11]. For example, does an organization need to code *all* their tests in LOINC or just their most commonly ordered tests to claim that they have implemented LOINC? Should the most recent version be used or are others acceptable? Are quality assurance processes required? Little is known/reported about how CTSA hubs have implemented or maintain LOINC codes in their systems and whether there is any variation that might impact the quality (accuracy and consistency) of LOINC-coded data for research.

## Methods

We developed a survey to assess operational dimensions of institutional LOINC implementation, maintenance, and use. The survey was developed by informaticians (TRC, DAH, BMK, RLR) from 3 CTSA sites (Cornell University, University of Michigan, University of Iowa). Most questions were multiple choice, with the option for free text comments to add context or to respond with answers not provided as options in the survey. The survey was introduced to potential participants at a CTSA informatics enterprise committee (iEC) meeting (most institutional informatics leads attend these monthly meetings) on May 3, 2024. A follow-up e-mail to iEC members asking for participation in the survey was sent later that day and once more on May 21, 2024. We requested one response per institution, and participants were encouraged to consult with others at their institution for additional details on their organizational LOINC implementation if needed. This study was reviewed by the Institutional Review Board (IRB) at the University of Michigan and determined to be exempt (IRB# HUM00249134). All data were reported as aggregate data in order to ensure that response data could not be used to identify a single institution. The full survey is provided in the Appendix.

## Results

Among the 64 CTSA-supported hubs, 19 (30%) responded to the survey and shared their experiences and approaches for implementing and maintaining LOINC. As seen in Table 1A, responding CTSAs use LOINC codes for a variety of purposes, with most sites (86%) using them for both local and networked research. About half the sites responded that LOINC was used for clinical care (47%) or quality initiatives (42%).

**Table 1.** Responses to selected survey questions. The questions are shown, as well as the number of sites that responded to the question (in parentheses). For all of the questions in this table, participants could select more than one answer. Percentages based on number of respondents for each item

(A) In what ways are LOINC codes used at your institution (19 sites responding)	
Local (institutional) research	16 (84%)
Networked research (e.g., Patient-Centered Outcomes Research Institute (PCORI))	16 (84%)
Clinical Care	9 (47%)
Quality Initiatives	8 (42%)
Public Health	7 (37%)
Data warehousing*	2 (11%)
Retrieving EHR data using FHIR*	1 (5%)
Unsure	0
(B) How do you allocate resources for routine LOINC mapping updates? (19 sites responding)	
No specific amount, “whatever it takes”	9 (47%)
Performed by non-CTSA team (e.g., IT operations, clinical lab)*	4 (21%)
Routine mapping updates not performed	3 (16%)
As needed for research requests*	3 (16%)
Effort allocation (Full-Time Equivalent (FTE)) per year	2 (11%)
NLP tool is used*	1 (5%)
Dollars per year	0
Hours per year	0
(C) What resources do you use to keep your LOINC mapping updated? (7 sites responding; % based upon 7 responses)	
Unified Medical Language System (UMLS)	5 (71%)
Regenstrief LOINC Mapping Assistant (RELMA®)	3 (42%)
Internal committees/collaborators	3 (42%)
Internally developed tool	3 (42%)
Online searches/chatbots	3 (42%)
EHR vendor	2 (29%)
Outside consultants	1 (14%)
OHDSI/Usagi mapping tool*	1 (14%)
Professional networks/list serves (e.g., asking others for advice)	0
Terminology management service (e.g., IMO, Apelon)	0

(D) What are the titles/roles/expertise of the individuals that carry out the LOINC mapping? (15 sites responding; % based upon 15 responses)	
Pathologist/laboratory expert	8 (53%)
Faculty	7 (47%)
Informatician	7 (47%)
Data analyst	5 (33%)
Terminology expert	4 (27%)
Developer	3 (20%)
Staff	2 (13%)
Vendor*	1 (7%)
Computational linguist*	1 (7%)
Research Informatics/Knowledge Engineering*	1 (7%)
Unsure	1 (7%)
Project manager	0
Trainees (student, post-doc, etc)	0
(E) Where does funding come from for maintaining LOINC? (15 sites responding; % based upon 15 responses)	
Health System	5 (33%)
No specific funding	5 (33%)
CTSA	3 (20%)
Unsure	3 (20%)
Departmental	2 (13%)
Institutional (research office, medical school, hospital)	2 (13%)
Start-up and research services cost recovery*	1 (7%)
Grants (not CTSA)	0

* Denotes write-in responses.

The reporting of allocation of resources for mapping to LOINC was also variable, as shown in Table 1B. Nearly half of the 19 respondents stated that they did not have a specific budget allocation for LOINC updates. 4 (21%) stated that LOINC mapping updates happened outside of CTSA budget (e.g., IT operations or clinical lab). 3 (16%) of responding CTSAs stated that routine mapping updates were not performed and the same amount 3(16%) responded that LOINC updates were performed as needed for research requests. Only 2 sites reported effort allocation for LOINC mapping updates. Only 3 sites felt that their organization provided sufficient funding; 5 sites reported not having enough funding, and 7 sites were unsure.

The version of LOINC reported to be used varied across sites. One site reported version 2.74; another site version 2.77; 9 sites reported using a mix of LOINC codes from different versions; and 3 respondents were unsure about the version of LOINC implemented at their institution. Respondents were asked what percentage of unique lab tests were mapped to LOINC at their institution. Four of the site respondents were unsure and did not provide an estimate. Among the other 15 sites, estimates ranged from 8 to 97%, with a mean of 74% of unique lab tests mapped. Of the 7 responses to frequency of updates for local (institutional) LOINC codes with the latest LOINC version, 3 reported variable frequencies, 1 yearly, 1 quarterly or semi-annually, 1 daily, and 1 unsure.

When asked if sites had formal/documented procedures for mapping LOINC, 3 (16%) reported having procedures, 5 (26%) reporting not having procedures, with the majority (11 sites; 58%) unsure. Among the 3 sites with formal procedures, all 3 responded that they were at least partially based on existing ISO/WHO guidelines [12]; 8 (42%) of responding institutions have processes to keep their LOINC mapping updated, 4 (21%) do not, and 7 (37%) were unsure if any processes existed. Only 7 sites responded to the question “*What resources do you use to keep your LOINC mapping updated?*” (Table 1C); of those, 5 (26%) reported that they used Unified Medical Language System (UMLS) and 3 (16%) reported use of Regenstrief LOINC Mapping Assistant (RELMA®). Eight other respondents reported various other resources, including internal committees/collaborators, locally developed mapping tools, online searches/chatbots, EHR vendor, outside consultants, and OHDSI/Usagi mapping tool. No sites reported using professional networks/list serves (e.g., asking others for advice) or terminology management services (e.g., Intelligent Medical Objects (IMO), Apelon).

Fifteen of the 19 sites provided a response to the question about the titles of individuals responsible for LOINC mapping at their institution; one of those reported “unsure.” The most frequently reported title was pathologist/laboratory expert (*n* = 8, 53%) followed by informatician (*n* = 7, 46%). Several different roles were entered as write-in responses (Table 1D).

## Discussion

In our survey of CTSA hubs, we observed substantial variability in both the level and types of approaches for LOINC implementation. There does not appear to be a uniform approach to implementing LOINC for research, raising concerns that variation in process might lead to variability in the assignment of codes and ultimately a lack of comparability. Further, there are no commonly reported metrics to quantify or estimate the completeness or quality of LOINC implementation at a given clinical research site, which limits opportunities for organizations to benchmark against or learn from sites with more “complete” LOINC implementations.

This survey was an exploratory step to understand LOINC practices in real-life institutional settings and to identify where there might be areas for shared learning. Organizations, including but not limited to CTSA hubs, wishing to begin or improve their LOINC implementation and ongoing maintenance would benefit from an understanding of the time, resources, and special expertise required to initiate and complete an implementation project. Moving forward, the research informatics community should define generalizable approaches to ensure broader consistency in how the codes are assigned and to evaluate the quality and consistency of these data for research. Additionally, the CTSA program might collaborate to identify efficiencies – perhaps through the sharing of expertise, methods, and tools across hubs – for implementing standardized coding systems as part of infrastructure to support national-level research initiatives.

A major limitation of this study is the low response rate. There is no way to know if 45 non-responding CTSA hubs have a consistent approach to LOINC implementation or if they have even implemented LOINC. It is possible that our results are not representative or generalizable across the entire CTSA program or academic medical centers. However, even in this small sample, we found significant variation on how a third of unique CTSA sites describe and approach their implementation of LOINC, which suggests the need for additional investigation and discussion on how to characterize, measure, and report LOINC adoption.

The lower adoption for clinical care reported by 9/19 (47%) sites is curious and somewhat surprising given the designation of LOINC by ONC for certified EHRs [3]. This might reflect a bias unawareness of use of LOINC in clinical care, as our respondents were informatics leaders in CTSA hubs focused primarily in research. These data require further investigation to interpret but do suggest an outstanding need for the biomedical informatics community to address native LOINC coding in clinical laboratory systems as well as an opportunity for closer collaboration between clinical IT and informatics [13].

As research informatics leaders, CTSA sites should be familiar with issues of information loss and error when mapping to standard coding systems [14]. Compliance with existing general [12,15] and LOINC-specific [16] mapping guidelines and documentation of implementation approaches should be ubiquitous, yet our findings suggest that there is variation and under-specification of methods, which could limit the comparability of data across sites and suggests possible issues with data quality or integrity for research. Previous informatics literature suggests that researchers establish standard processes for mapping LOINC and examining the quality of coding [9,17]. A recent American Medical Informatics Association (AMIA) podium abstract describes a dashboard for looking at LOINC data over time and underscores the needs for tools to support the quality of LOINC implementation and updates [18]. The CTSA program and national research infrastructure would benefit from well-specified and standardized practices for LOINC implementation and maintenance. These methods and reporting practices can inform implementation and use of other standardized coding systems, e.g., SNOMED-CT, used in networked research.

## Conclusion

There is substantial variability in approaches for LOINC implementation and updates across CTSA hubs that might have negative implications for data comparability and multi-site research. Standard methods and reporting for LOINC implementation could be used to assess research maturity and improve organizational readiness for networked research.

## Study highlights

What is the current knowledge on the topic? There is no best practice or uniform guidance for research sites to implement and maintain standardized coding systems. Additionally, there is no information on the types of personnel and other costs associated with LOINC implementation.

What question did this study address? This study asked research informatics leaders to share their technical and staffing approaches and experience with LOINC implementation to see if there are common approaches or requirements across sites.

What does this study add to our knowledge? We could not find a common approach for implementing LOINC coding in clinical research sites. This suggests a need for further communication and cooperation to define implementation and maintenance activities that will result in consistent and accurate coding.

How might this change clinical pharmacology or translational science? The development of standard approaches to collecting and sharing clinical and laboratory data used for research is a critical to assess and ensure the quality of data used for research analyses.

## Supporting information

10.1017/cts.2025.10151.sm001 Richesson et al. supplementary material Richesson et al. supplementary material

## Supplementary material

For supplementary material/s referred to in this article, please visit https://doi.org/10.1017/cts.2025.10151.
