# Supplementary material for: LOINC implementation approaches in academic medical research centers – results from a survey of CTSA sites
Source: J Clin Transl Sci. 2025 Sep 29;9(1):e223. doi: 10.1017/cts.2025.10151 (PMC12529624; doi:10.1017/cts.2025.10151)
Supplement: Richesson et al. supplementary material [file S2059866125101519sup001.pdf]

Tools Saved Apr 23, 2024 at 9:49 AM Live Preview Publish

LOINC-CTSA

ExpertReview score Great

Default Question Block

Q1

**Operational LOINC Survey**

Thank you for participating in this LOINC survey. We are asking that one representative from each institution answer these questions. Feel free to consult with others at your institution as needed to complete this survey.

The purpose of this survey is to gain a better understanding of operational aspects of maintaining local data mapped to LOINC. It is entirely voluntary and you do not have to complete the survey if you are not comfortable answering these questions. This survey has been reviewed and determined to be exempt by the University of Michigan IRB (IRB# HUM00249134). If you have any concerns, please contact us: David Hanauer (hanauer@umich.edu) or Rachel Richesson (richessr@med.umich.edu).

Q2

Name of person completing this survey
(This will be kept confidential and is only being asked in case we have follow-up questions)

Q12

Email of person completing this survey
(This will be kept confidential and is only being asked in case we have follow-up questions)

Q4

May we include the name of your institution as a participant in this survey? (Institution names tied to specific results will not be reported, but we would like to report which institutions participated.)

- Yes
- No

Q5

Display this question

If May we include the name of your institution as a participant in this survey? (Institution names t... Yes Is Selected

Name of institution/site

Q6

In what area(s) do you work at your institution (check all that apply):

- Academic Department
- Clinical IT
- CTSA/CTSI
- EHR Team
- Informatics
- Information Technology (IT)
- Pathology/laboratory
- Research Office
- Other (please type response):

Q7 *

In what ways are LOINC codes used at your institution (check all that apply):

- ☐ Local research within your institution
- ☐ Networked research (e.g., PCORnet or other multi-institutional collaborations)
- ☐ Clinical care
- ☐ Quality initiatives
- ☐ Public Health
- ☐ Unsure
- ☐ Other (please type response):

Q8 *

Approximately what percentage of your lab data are mapped to LOINC?
*(Base your estimate on the number of distinct lab tests you have and how many of these distinct test are mapped to a LOINC code)*

- ○ Percentage (enter whole number between 0 and 100):
- ○ Unsure

Q9 *

How do you allocate resources for routine LOINC mapping updates? Please specify approximately how much is allocated per year.

- ☐ FTE per year:
- ☐ $ per year:
- ☐ hours per year:
- ☐ No specific amount, whatever it takes
- ☐ Routine updates not performed
- ☐ Other (please explain):

Q10 *

Does your institution have formal/documented procedures for mapping LOINC codes?

- Yes
- No
- Unsure

Q11 *

Display this question

If Does your institution have formal/documented procedures for mapping LOINC codes? Yes Is Selected

Are your formal/documented procedures based, at least in part, upon existing ISO/WHO guidelines? (For example: ISO, WHO)

- Yes
- No
- Unsure

Q13 *

Do you have processes to keep your local LOINC mapping updated?

- Yes
- No
- Unsure

Q14 *

Display this question

If Do you have processes to keep your local LOINC mapping updated? Yes Is Selected

What resources do you use to keep your LOINC mapping updated? (check all that apply):

- ☐ EHR vendor
- ☐ Internal committees/collaborators
- ☐ Internally developed tool
- ☐ Online searches/chatbots
- ☐ Outside consultants
- ☐ Professional networks/list serves (e.g., asking others for advice)
- ☐ Regenstrief LOINC Mapping Assistant (RELMA®)
- ☐ Terminology management service (e.g., IMO, Apelon)
- ☐ Unified Medical Language System (UMLS)
- ☐ Other (please specify):

Q15 *

Display this question

If Do you have processes to keep your local LOINC mapping updated? Yes Is Selected

Approximately what is the frequency with which your local LOINC codes are updated to be current with the latest LOINC data?

- ○ Daily
- ○ Weekly
- ○ Monthly
- ○ Quarterly or semi-annually
- ○ Yearly
- ○ Variable, e.g., upon request or identification of invalid mappings (e.g., through data quality checks from national networks)
- ○ Not routinely updated
- ○ Unsure

Q16 *

What are the titles/roles/expertise of the individuals that carry out the LOINC mapping (check all that apply; a single individual can be assigned to more than one category).

- ☐ Data analyst
- ☐ Developer
- ☐ Faculty
- ☐ Informatician
- ☐ Pathologist/laboratory expert
- ☐ Project manager
- ☐ Staff
- ☐ Terminology expert
- ☐ Trainees (student, post-doc, etc)
- ☐ Unsure
- ☐ Other (please specify): ______

Q17 *

What version of LOINC is your institution currently using?

- ○ Write in: ______
- ○ We are using a mix of LOINC codes from different versions
- ○ Unsure

Q18 *

Where does funding come from for maintaining LOINC? (check all that apply)

- ☐ Departmental
- ☐ CTSA
- ☐ Grants (not CTSA)
- ☐ Health System
- ☐ Institutional (research office, medical school, hospital)
- ☐ No specific funding
- ☐ Unsure
- ☐ Other: ______

Q19

In your opinion, does your organization provide sufficient funding for LOINC implementation and maintenance/upkeep/updates/monitoring?

- Yes
- No
- Unsure

Q21

At your site, how is adjudication done between codes that might seem like they can equally apply?

Q22

Does your site offer researchers the ability to access lab data mapped to LOINC through a self service tool?

- Yes, and the tool is:
- No
- Unsure

Q23

Approximately how many individuals or research teams use/request LOINC data for research per year?

Q24

Which research networks/collaborations does your institution participate in (select all that apply):

- [ ] 4CE
- [ ] ACT/ENACT
- [ ] Cerner Real World Data
- [ ] Epic Cosmos
- [ ] N3C
- [ ] OMOP/OHDSI
- [ ] PCORnet
- [ ] Sentinel
- [ ] TriNetX
- [ ] Other (please specify):

Q25

Please let us know if you have any additional thoughts or comments:

Import from library

Add new question

Add Block

End of Survey

We thank you for your time spent taking this survey.

Your response has been recorded.
